# A multi-centre study of belatacept and sirolimus for islet transplantation

**DOI:** 10.1007/s00125-026-06813-3

**Published:** 2026-08-17

**Authors:** Natasha M. Rogers, Min Hu, Elvira Jimenez-Vera, Hugh Gabor, Preeti P. Sharma, Colleen Etherton, Tom Loudovaris, Wayne J. Hawthorne, Chris J. Drogemuller, David J. Torpy, Deborah J. Holmes-Walker, Brian Gloss, Shane T. Grey, David Goodman, P. Toby Coates, Tom W. Kay, Philip J. O’Connell

**Affiliations:** 1 National Pancreas Transplant Unit, Westmead Hospital, Westmead, NSW Australia; 2 Centre for Transplant and Renal Research, Westmead Institute for Medical Research, Westmead, NSW Australia; 3 Faculty of Medicine and Health, The University of Sydney, Camperdown, NSW Australia; 4 Renal and Transplantation Medicine, Westmead Hospital, Westmead, NSW Australia; 5 Central Northern Adelaide Renal and Transplantation Service, Royal Adelaide Hospital, Adelaide, SA Australia; 6 Immunology and Diabetes Unit, St Vincent’s Institute of Medical Research, Melbourne, VIC Australia; 7 Endocrine and Metabolic Unit, Royal Adelaide Hospital, Adelaide, SA Australia; 8 Department of Diabetes and Endocrinology, Westmead Hospital, Sydney, NSW Australia; 9 Westmead Bioinformatics Facility, Westmead Institute for Medical Research, Westmead, NSW Australia; 10 School of Biotechnology and Biomolecular Sciences, Faculty of Science, The University of New South Wales, Sydney, NSW Australia; 11 Department of Nephrology, St Vincent’s Hospital Melbourne, Fitzroy, VIC Australia; 12 Department of Medicine, The University of Melbourne, Parkville, VIC Australia

**Keywords:** Belatacept, Calcineurin inhibitor, Immunosuppression, Islet transplantation, Nephropathy, Regulatory T cells, Sirolimus, Type 1 diabetes

## Abstract

**Aims/hypothesis:**

Islet transplantation is an appropriate treatment for selected individuals with type 1 diabetes mellitus and hypoglycaemia unawareness. The gold standard of maintenance immunosuppression to prevent allograft rejection is calcineurin inhibitor-based therapy; however, side effects warrant exploration of alternative immunosuppression. The co-stimulatory blocker belatacept and the mammalian target of rapamycin inhibitor sirolimus have demonstrated clinical benefit in solid organ transplantation, but efficacy in human islet transplantation is unknown.

**Methods:**

We conducted a non-randomised, phase 2, multi-centre, open-label clinical study. Participants with type 1 diabetes receiving at least one islet transplant were treated with belatacept/sirolimus (bela/siro) and compared with a contemporaneous cohort treated with tacrolimus/mycophenolate mofetil (tac/MMF). The primary outcome was freedom from hypoglycaemia, with positive C-peptide and HbA_1c_ <53 mmol/mol (7.0%) at 12 months after the first transplant. Secondary outcomes included metabolic control (Igls criteria, BETA-2 score), allosensitisation and changes in peripheral blood immune cell populations.

**Results:**

Nine participants with type 1 diabetes received bela/siro, compared with 24 receiving tac/MMF. Eight participants (89%) receiving bela/siro achieved the primary outcome compared with 12 (52%) from the tac/MMF cohort. HbA_1c_, HYPO score and insulin requirements were reduced in both groups, although renal function remained unchanged only in the bela/siro group. Immunosuppression resulted in sustained suppression of all monocyte, NK, T and B cell subsets, although CD4^+^FOXP3^+^ regulatory T cell proportions were higher in the bela/siro group.

**Conclusions/interpretation:**

Baseline immunosuppression with bela/siro facilitated early islet engraftment and rates of insulin independence, with less impact on renal function, supporting the rationale for routine use in islet transplantation.

**Trial registration:**

ANZCTR.org.au ACTRN12619000268145

**Graphical Abstract:**

**Figure:**
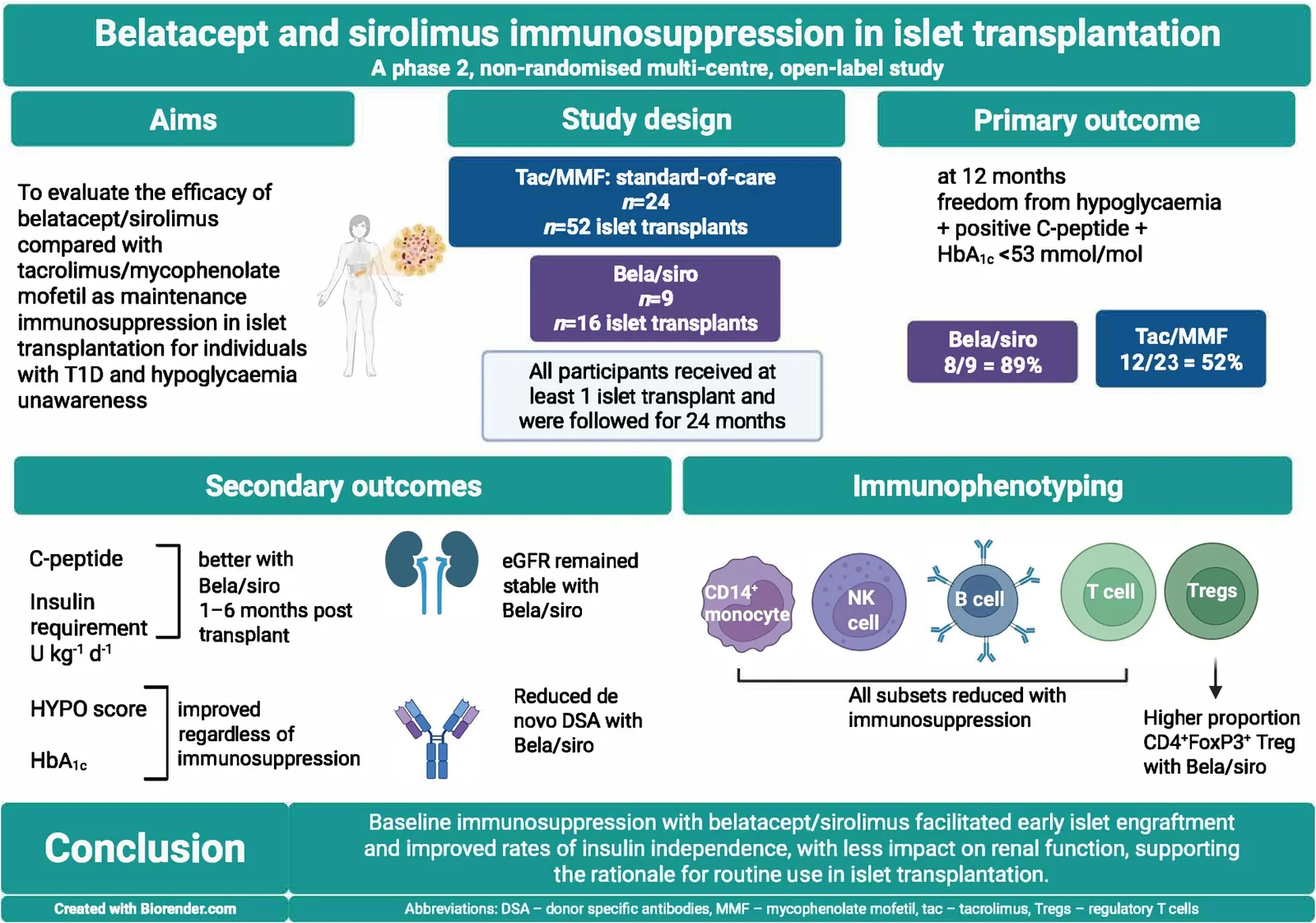

**Supplementary Information:**

The online version contains peer-reviewed but unedited supplementary material available at https://doi.org/10.1007/s00125-026-06813-3.

## Introduction

**Figure:**
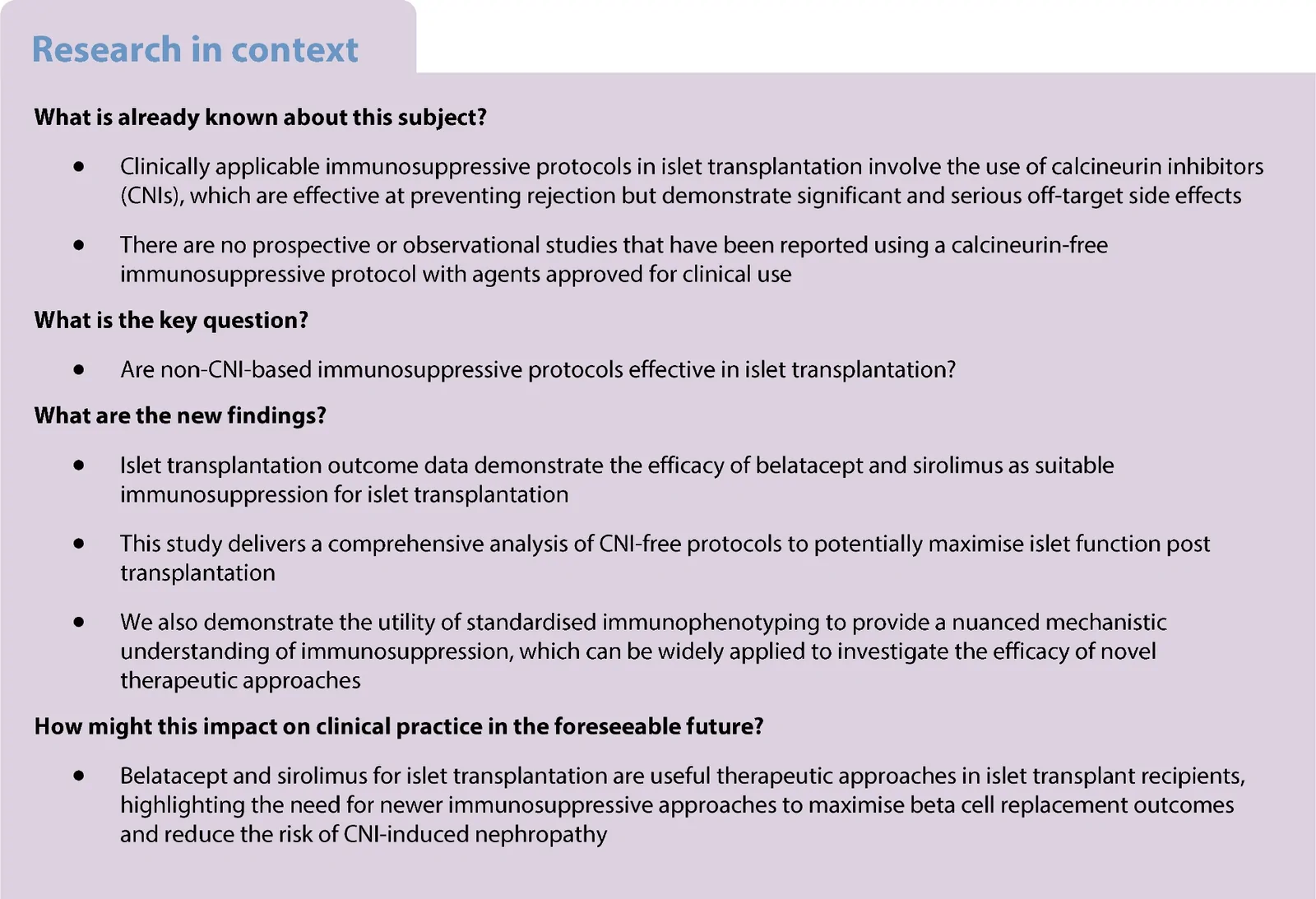

Clinical islet transplantation is an established procedure with well-documented efficacy for individuals with type 1 diabetes mellitus and impaired awareness of hypoglycaemia (IAH) accompanied by severe hypoglycaemic events (SHE). Long-term rates of insulin independence and glycaemic control are inferior to those seen with whole pancreas transplantation [1], thought to be due to lower engrafted islet mass after losses during isolation and initial destruction due to delayed revascularisation [2]. Islet transplantation is accepted practice when kidney transplantation is not required or when recipients are not fit for whole pancreas transplantation. Reduced longevity of islet transplantation is offset by minimally invasive delivery, lower peri-operative morbidity and the ability to maximise beta cell replacement with multiple transplants.

Immunosuppression for the life of an allograft remains dependent on calcineurin inhibitors (CNIs), which adversely affect beta cell mass [3], promote insulin resistance [4] and impair renal function following non-renal solid organ transplantation [5]. The co-stimulator blocker belatacept represents a significant advance in calcineurin-free immunosuppression, with discernible preservation of renal function in kidney [6], heart [7] and lung [8] transplant trials, as well as absent metabolic effects [9]. The mammalian target of rapamycin (mTOR) inhibitor sirolimus was part of the original Edmonton protocol [10], providing an additional, unique benefit with the selective induction and expansion of CD4^+^CD25^+^FOXP3^+^ regulatory T cells (Tregs), which has been replicated in mice [11] and humans [12]. Although belatacept does not explicitly promote generation of Tregs, it may be permissive in maintaining (peripheral) Treg numbers [13]. Recent studies in non-human primates have demonstrated significant prolongation of islet transplant survival using belatacept and sirolimus [14]. Although a role for Tregs was not implicated in either study, belatacept-treated animals failed to generate anti-donor responses during treatment.

Here we report a phase 2 trial of allogeneic islet transplantation in individuals with type 1 diabetes conducted by the Australian Islet Consortium. We compared belatacept in combination with sirolimus (bela/siro) with standard-of-care immunosuppression (tacrolimus/mycophenolate mofetil, tac/MMF), and participants were followed for a minimum of 12 months. Our goal was to explore clinical efficacy and metabolic outcomes, as well as inherent risks of islet transplantation, including CNI-induced nephropathy and allosensitisation, and how these may be modified by immunosuppression. We complemented these biochemical parameters with a comprehensive immunophenotyping assessment which was used to address our hypothesis of modulated recipient immune reactivity and extend mechanistic understanding in relation to differing clinical outcomes with co-stimulatory blockade and mTOR inhibition.

## Methods

### Study population

This was a prospective, single-arm, multi-centre, open-label, non-randomised study of bela/siro in pancreatic islet transplantation for adult individuals with type 1 diabetes and concomitant SHE with IAH. The trial was designed to evaluate the feasibility, safety and immunological effects of bela/siro. These participants were compared with a reference standard-of-care group receiving tac/MMF. All participants receiving bela/siro were enrolled and transplanted from February 2019 through to December 2023. Participants receiving tac/MMF were concurrent control individuals from the Australian Islet Transplant Consortium enrolled and transplanted between January 2015 and December 2023.

The study was designed and reported in compliance with the Consolidated Standards of Reporting Trials (CONSORT) 2010 statement. All participants met study inclusion and exclusion criteria. Eligibility criteria included age >18 years and a diagnosis of type 1 diabetes >5 years at time of assessment. Clinical severity of hypoglycaemia was graded using the validated Edmonton HYPO score [15], where a score of >1047 (90th percentile) was considered indicative of severe hypoglycaemia unawareness, or alternatively a Clarke Score >4 where participants were using continuous glucose monitoring to reduce frequency of hypoglycaemia. Participants were free of diabetic nephropathy (proteinuria <300 mg/day) and renal impairment (glomerular filtration rate [GFR] >75 ml/min per 1.73 m^2^). Three participants were islet-after-kidney transplant recipients with stable renal allograft function. All participants gave written informed consent, and the protocol was approved by the individual Human Research Ethics Committees of the three participating Australian institutions (Western Sydney Local Health District HRECs 2006/34/4.5-2305, 2019/ETH02082/PID02425 and 2020/ETH02188/PID02435; St Vincent’s Hospital, Melbourne, SVH HREC-D103/05; Royal Adelaide Hospital, Adelaide, MyIP10845). The trial was overseen by Westmead Hospital Islet Transplant Unit and registered with the Australian and New Zealand Clinical Trials Registry (ANZCTR registration no. ACTRN12619000268145, https://anzctr.org.au/Trial/Registration/TrialReview.aspx?id=375823).

### Islet preparation and transportation

Islets were separated at two islet isolation facilities using a variation of the closed-loop method [16] which was standardised across both centres. Pancreases were removed from donation after brain death donors (DBDD) and disaggregated by infusing the ducts with Collagenase NB 1 GMP Grade and Neutral Protease NB GMP Grade (SERVA Electrophoresis, Germany). Dissociated islet and acinar tissues were separated on a continuous Biocoll (Biochrom, Germany) density gradient (polysucrose 400 and amidotrizoic acid) on a refrigerated apheresis system (Model 2991, COBE Laboratories, Lakewood, CO). Purified islets were counted, and islet number and mass were expressed in terms of islet equivalents (IEQ). Preparations underwent a pre-transplant quality assurance process, including a Gram stain, purity and viability assessment, packed cell volume measurement and evaluation of islet morphology to exclude excessive fragmentation. Islets were cultured in Miami media in 95% room air and 5% CO_2_ at 37°C for up to 24 h.

### Islet transplantation

Islets were resuspended in 100 ml in CMRL1066 (Nucleus Biologics, San Diego, CA) containing 5000 U heparin and 20% human albumin. Islets were transplanted either at the local facility where the isolation was undertaken, or in Adelaide (730 km from Melbourne and 1400 km from Sydney). Islets were transported by air in 100 ml in CMRL1066 (Nucleus Biologics) and 4% human albumin (CSL, Australia) at ambient temperature and transplanted immediately upon arrival. At the Royal Adelaide Hospital and St Vincent’s Hospital, the portal vein was accessed via a percutaneous transhepatic approach. At Westmead Hospital, either a mini-laparotomy (until 2020) or percutaneous hepatic approach was used. For the latter procedure, an arterial angiographic catheter was inserted into the main portal vein with the assistance of image intensification. Hepatic portal pressure was measured at 5 min intervals during infusion. There was no mandated time for subsequent islet infusions to occur. The shortest time between infusions was 28 days and the longest 137 months.

### Immunosuppression

Immunosuppression was instituted at the time of transplantation, and consisted of rabbit antithymocyte globulin (ATG, Sanofi-Aventis, Australia) for the first two transplants (at doses of 4.5 mg/kg and 3 mg/kg, respectively), with basiliximab (20 mg) replacing ATG for the third transplant. Participants in the standard-of-care group (tac/MMF) received tacrolimus (Astellas, Japan) commenced at 0.1 mg kg^−1^ day^−1^ in two divided doses to achieve a target blood concentration of 10–12 ng/ml. MMF [9] was prescribed at a dose of 1 g twice a day. Etanercept (Pfizer, NY) was given subcutaneously (50 mg on day 0, 25 mg on days 3, 7 and 10). The bela/siro group received 10 mg/kg of belatacept (provided by Bristol Myers Squibb, NY) on days 1, 5, 15 and 28, then monthly until week 12, and 5 mg/kg monthly thereafter. Second and subsequent transplants received 5 mg/kg on day 1, fortnightly dosing for weeks 2–8, then monthly administration. Sirolimus (Pfizer) was commenced at a dose of 5 mg daily, with the dose adjusted to achieve a target blood concentration of 3–7 ng/ml. Participants were also commenced on the dipeptidyl peptidase 4 inhibitor sitagliptin (Merck Sharpe and Dohme, Whitehouse Station, NJ) and/or extended-release metformin (Diabex XR, Alphapharm, Australia) as tolerated. The dosing schedule is shown in electronic supplementary material (ESM) Table 1.

### Post-transplant monitoring

Graft function was assessed with blood glucose monitoring (finger stick or continuous glucose monitoring), HbA_1c_, fasting glucose and C-peptide. The primary outcome was a composite measure of freedom from hypoglycaemia (calculated by the Edmonton HYPO score), the percentage of participants who were C-peptide positive with HbA_1c_ <53 mmol/mol (7.0%) at 12 months from first transplant. Secondary outcomes included the proportion of recipients that were insulin independent (requiring <5 U/day) for more than 1–2 weeks, overall metabolic control (HbA_1c_, C-peptide) and allosensitisation. There was no change in the maximum number of islet infusions permitted (up to three). Safety data were reviewed by an independent and external data and safety monitoring board on an ongoing basis.

### Immunologic screening and immunophenotyping analysis

Participants provided whole blood samples, collected pre-transplantation and at pre-determined post-transplant intervals (0.5, 1, 3 and 12 months) into EDTA vacutainers (BD Biosciences, Franklin Lakes, NJ), and 1.5 ml was used in the study. All samples were stained within 24 h of collection. Flow cytometric immunophenotyping was performed in keeping with our previously published, standardised clinical trial protocol [17]. Antibody clones, concentrations, staining procedures and cell count analyses were used as described previously [17]. All antibodies were obtained from BD Biosciences, except CCR7 (R&D Systems, Minneapolis, MN). In brief, four panels were used to determine six absolute cell populations, including CD14⁺ monocytes, CD19^+^ B cells, CD56⁺ NK cells, CD3⁺ T cells, CD3⁺CD56⁺ NKT cells, CD4⁺FOXP3⁺ Tregs and relevant subsets. The three main staining protocols used were [17]:

1. Absolute cell number panel: Anticoagulated whole blood was stained in Trucount tubes with Fc block and antibody cocktail, then lysed/fixed using BD FACS Lysing Solution.
2. T and B cell panels: Anticoagulated whole blood was Fc-blocked, stained with antibody cocktail, lysed/fixed and washed before acquisition; the B cell panel included an additional Dulbecco’s phosphate-buffered saline (DPBS) wash step before Fc-blocking.
3. CD4⁺FOXP3⁺ Treg panel: Surface staining was performed as above, followed by fixation, permeabilisation (BD Transcription Factor Buffer Set) and intracellular FOXP3 staining before acquisition.

Samples were acquired on a BD LSRFortessa flow cytometer, and data were analysed using FlowJo software (v11.2, https://www.flowjo.com/flowjo/download, FlowJo, Ashland, OR).

For subset identification, standardised gating strategies were used to define CD19⁺ B cells, CD4⁺ and CD8⁺ T cells, and CD4⁺FOXP3⁺ Tregs in each relevant panel, as previously described [17]. Gated events and channel values were then imported into R using flowWorkspace (v4.22.1, https://www.bioconductor.org/packages/release/bioc/html/flowWorkspace.html) and analysed with the Spectre package. The gating hierarchy was used to isolate the relevant cell populations, after which marker intensities were rescaled and subjected to FlowSOM clustering with meta-*k* values ranging from 10 to 40. FlowSOM metaclusters were manually assigned to final clusters based on marker expression profiles and manual gating information. A subset of data containing an equal number of cells from each group was used for Uniform Manifold Approximation and Projection (UMAP). UMAP plots were generated either as combined visualisations or as density plots for each group. We performed UMAPs of 50,000 cells (with equal numbers from each drug group), coloured according to manual clusters, with manual gating (terminal gate) or cell-density distribution, depending on the panel shown.

### Sex and gender considerations

Sex was considered in the study design through the inclusion of both male and female participants, and self-reported participant sex was recorded at enrolment. The study was not specifically designed or powered to detect sex-based differences in clinical outcomes, and no sex-stratified analyses were prespecified. Gender identity was not collected as a study variable.

### Statistics

Continuous variables are expressed as mean ± SD (with minimum–maximum ranges) or median ± interquartile range as stated, and data were analysed using GraphPad Prism (v10.2.3, GraphPad Software, Irvine, CA); *p*<0.05 was considered significant. Pre-transplant donor and recipient characteristics were compared with Mann–Whitney *U* test (non-normally distributed data) or unpaired *t* test for normally distributed data (a Welch’s *t* test was applied for unequal variances). Categorical variables were presented as frequencies and percentages, compared using χ^2^ or Fisher’s exact test. Changes in pre- and post-transplantation metabolic outcomes were analysed using a linear mixed-effects model considering time and group effects. Missing variables are documented in ESM Table 2. For immunophenotyping results, cellular proportions and counts were analysed in SPSS (IBM, Chicago, IL). Normality was assessed with the Shapiro–Wilk test. Longitudinal comparisons between the two groups were performed at each timepoint using the Mann–Whitney *U* test, and within-group comparisons between pre-treatment and post-transplantation timepoints using the Wilcoxon signed-rank test.

## Results

### Recipient and donor characteristics

Between January 2015 and December 2023, 24 individuals with type 1 diabetes received tac/MMF with 52 islet transplants (*n*=9 received 3 transplants, *n*=10 received 2 transplants and *n*=5 received 1 transplant). Between March 2019 and December 2023, nine individuals received bela/siro for 16 islet transplants (*n*=2 received 3 transplants, *n*=3 received 2 transplants and *n*=4 received 1 transplant, *p*=0.44 compared with tac/MMF group, Fisher’s exact test). Donor graft characteristics are presented in Table 1, with no significant differences between the groups.

**Table 1:** Donor characteristics

Characteristic	Tac/MMF (*n*=52) Mean ± SD Minimum–maximum	Bela/siro (*n*=16) Mean ± SD Minimum–maximum	*p* value
Age (years)	47.5±10.4 24–69	44.6±12.1 20–58	*p*=0.40
Sex			*p*=0.46
M	27 (52)	10 (62.5)	
F	25 (48)	6 (37.5)	
Weight (kg)	91.9±20.3 48–170	102.6±21.5 70–140	*p*=0.091
BMI (kg/m^2^)	30.8±6.1 19.2–47.6	31.3±5.7 25–48.4	*p*=0.75
Cold ischaemic time (min)	348±110.3 102–584	390±149 200–667	*p*=0.31
IEQ isolated	500,720±178,307 263,255–1,211,349	462,892±106,707 300,511–725,988	*p*=0.31
IEQ/g pancreas	5293±2299 2691–13,459	4650±1138 2639–7192	*p*=0.14
Purity (%)	78.5±14.7 20–95	71.2±16.9 30–90	*p*=0.13
Viability (%)	88.1±7 65–100	90.7±5.1 80.5–95	*p*=0.12

Data are presented as *n* (%) or mean ± SD with minimum-to-maximum values

The baseline characteristics of the recipients are shown in Table 2, with no significant differences between cohorts. The mean age of tac/MMF and bela/siro recipients, respectively, was 48.8±12 vs 51.6±11.7 years (*p*=0.55), BMI was 24.3±3.3 vs 25.2±3.3 kg/m^2^ (*p*=0.47) and duration of diabetes prior to transplantation was 33.3±12.4 vs 26±4.7 years (*p*=0.074). There was no sex distribution difference (50% and 56% female in the tac/MMF and bela/siro cohorts, respectively). All recipients had severe hypoglycaemia, with 55% of recipients from both groups having HYPO scores >1047 at transplantation. The remaining recipients had a HYPO score <1000 as a result of increased blood glucose monitoring but otherwise fulfilled the criteria for hypoglycaemia, with IAH and ≥1 SHE in the 12 months prior to study enrolment. Baseline autoantibody titres are shown in Table 2; approximately 88% of the tac/MMF and 67% of the bela/siro cohort had autoantibodies directed against glutamic acid decarboxylase-65 (GAD65), with 67% and 33%, respectively, having measurable islet antigen-2 (IA-2) antibodies. Three participants from the tac/MMF cohort received an islet-after-kidney transplant; the remaining were islet transplants alone.

**Table 2:** Recipient characteristics at first islet transplant

Characteristic	Tac/MMF (*n*=24) Mean ± SD Minimum–maximum	Bela/siro (*n*=9) Mean ± SD Minimum–maximum	*p* value
Age (years)	48.8±12 28.4–66.2	51.6±11.7 32–66	*p*=0.55
Sex			*p*=0.99
M	12 (50)	4 (44)	
F	12 (50)	5 (56)	
BMI (kg/m^2^)	24.3±3.3 15.3–32.7	25.2±3.3 18.7–28.8	*p*=0.47
Weight (kg)	71.0±12 39.2–92.5	71.6±12.1 49.1–91.2	*p*=0.94
Duration of diabetes (years)	33.3±12.4 12.2–53.2	26±4.7 19.8–31.5	*p*=0.074
HbA_1c_ (mmol/mol)	68.4±16.2 44.3–124	60.8±12.6 44.3–80.34	
			*p*=0.19
HbA_1c_ (%)	8.4±1.5 6.2–13.5	7.7±1.1 6.2–9.5	*p*=0.19
Insulin requirements (U/day)	37.7±15.1 14–72	33.8±11.1 22.3–54	
			*p*=0.40
Insulin requirements (U kg^−1^ day^−1^)	0.53±0.24 0.11–1.41	0.47±0.102 0.32–0.59	*p*=0.29
Autoantibodies (% positive)			
Anti-GAD65	21/24 (87.5)	6/9 (67)	*p*=0.31
Anti-IA-2	16/24 (67)	3/9 (33)	*p*=0.12
Edmonton HYPO score	1784±1986 60–7771	2115±2069 304–6236	*p*=0.81
eGFR (ml/min per 1.73 m^2^)^a^	101±19.5 65–121	97.4±11.3 81–119	*p*=0.14
No. of transplants			*p*=0.20
1	5 (20.8)	4 (44.4)	
2	10 (41.7)	3 (33.3)	
3	9 (37.5)	2 (22.2)	
Total transplanted IEQ per transplant	403,076±117,286 197,371–922,234	364,101±67,451 256,012–508,597	*p*=0.21
IEQ/kg per transplant	5956±2088 3289–16,180	5081±995 3649–7058	*p*=0.11
IEQ/kg total	13,281±5216 3289–24,057	9033±3684 5268–16,213	*p*=0.034
Time to subsequent transplant (days)	481±475 15–1646	949±726 22–2353	*p*=0.07

Data are presented as *n* (%) or mean ± SD with minimum-to-maximum values

^a^Estimated by the Chronic Kidney Disease Epidemiology Collaboration (CKD-EPI) equation, excluding three IAK participants

Fifty out of 68 islet transplants (74%) were performed at the centre of isolation, with 18 islet preparations isolated at a distant centre (*n*=13/50 for tac/MMF, and *n*=5/16 for bela/siro recipients). Islets were infused via interventional radiology and the transhepatic approach in all participants receiving bela/siro and via mini-laparotomy in two participants (8.3%) receiving tac/MMF. There were no differences in the total IEQ or IEQ/kg per transplant infused between groups, although total IEQ/kg was lower in the bela/siro group (13,281±5216 vs 9033±3684, *p*=0.034).

### Clinical and metabolic outcomes after islet transplantation

Twenty (83%) individuals enrolled in the tac/MMF study and nine individuals (100%) from the bela/siro cohort completed >12 months of evaluation from the time of the first islet transplant. Four individuals from the tac/MMF arm withdrew from the study (one at 3 months, and three at 12 months post transplant), leading to cessation of immunosuppression and allograft failure. At 12 months, 52% of tac/MMF participants and 89% of bela/siro participants reached the primary outcome (*p*=0.103, Fisher’s exact test).

### Igls criteria and BETA-2 score

**Fig. 1:**
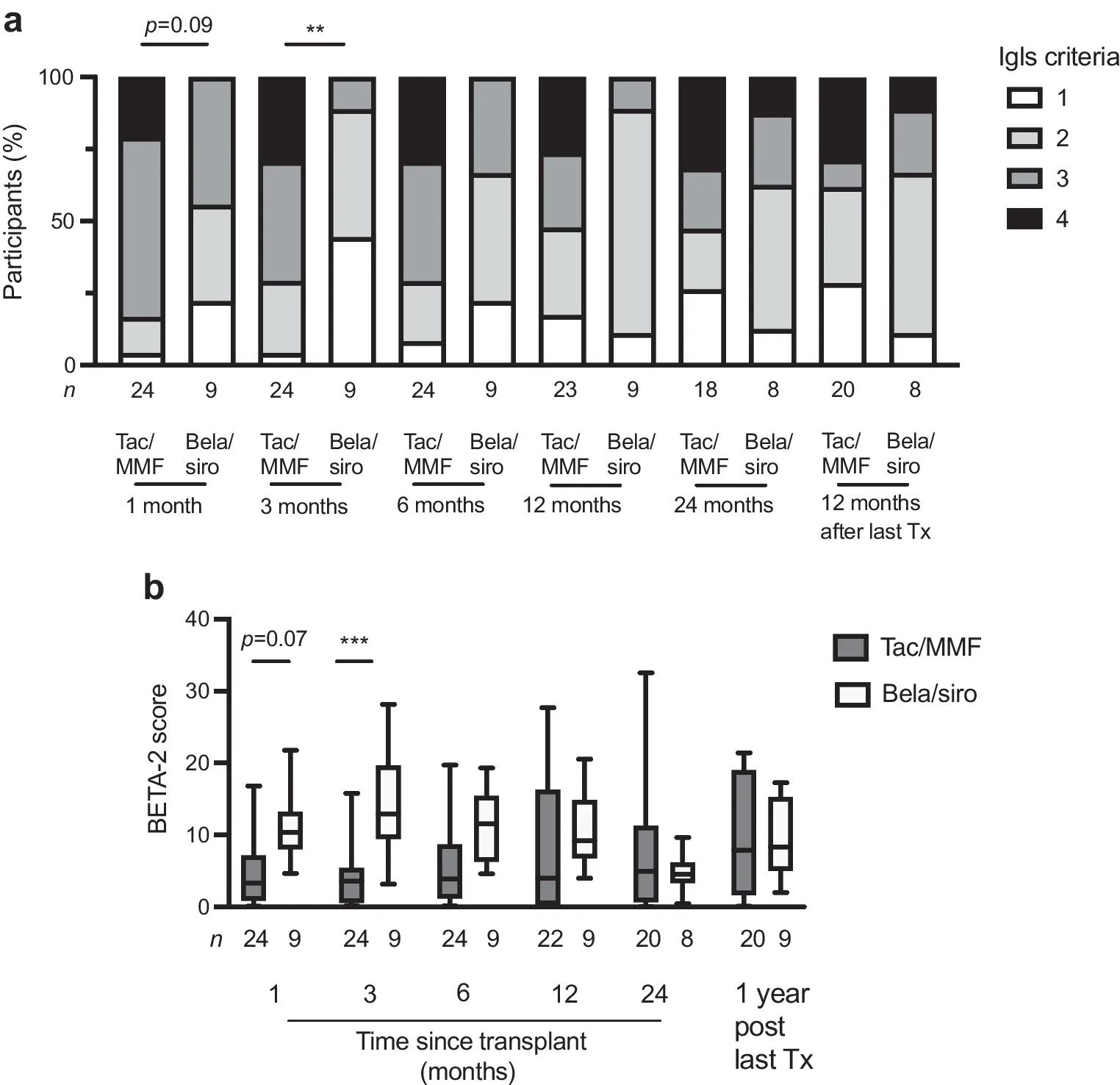
Igls criteria- and BETA-2 score-based outcomes after islet transplantation. (**a**) Igls criteria and (**b**) BETA-2 score were used as a standardised measure of allograft function. Assessments were performed at 1, 3, 6, 12 and 24 months after the first transplant and 12 months after the last transplant. (**a**) Bars represent the percentage of participants in each Igls category (1 = optimal, 2 = good, 3 = marginal, 4 = failed graft function). (**b**) BETA-2 scores are presented as median, interquartile range and minimum-to-maximum values. (**a**, **b**) The *p* values were derived from Fisher’s exact test, comparing distributions between bela/siro and tac/MMF immunosuppression at each timepoint: **p<0.01, ***p<0.001. Tx, treatment

We used the Igls criteria [18] (1 = optimal, 2 = good, 3 = marginal, 4 = failed graft function) as a primary trial outcome, which provides a composite analysis of SHE, C-peptide, HbA_1c_ and insulin use, and reflects the capacity to compare multiple clinically relevant data. The distribution of scores differed between the two immunosuppression regimens over time (Fig. 1a). Participants treated with tac/MMF were more likely to experience marginal or failure of graft function at 3 months compared with those receiving bela/siro (*p*=0.006, Fisher’s exact test). However, this difference was not maintained at other timepoints after the first islet transplant (*p*=0.094, *p*=0.142, *p*=0.10 and *p*=0.38 at 1, 6, 12 and 24 months, respectively), as well as at 12 months after the last transplant (*p*=0.48). Similar differences were seen in the BETA-2 score [19] (Fig. 1b).

**Fig. 2:**
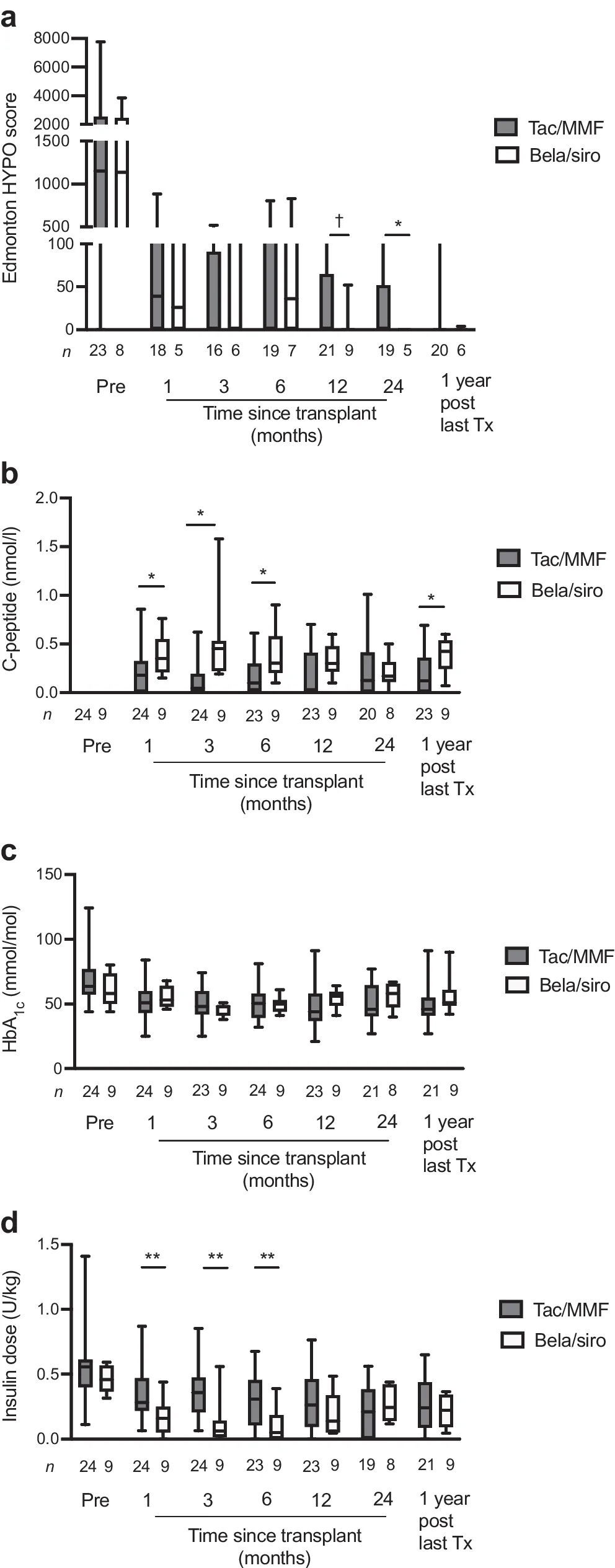
Metabolic outcomes after islet transplantation. (**a**) HYPO scores, (**b**) C-peptide (nmol/l), (**c**) HbA_1c_ (mmol/mol) and (**d**) insulin requirements (U/kg) pre-transplantation, 1, 3, 6, 12 and 24 months after the first islet transplant and 12 months after the last transplant in participants receiving bela/siro or tac/MMF immunosuppression. Longitudinal and between-group comparisons were performed using a mixed-model effect. Results are reported as median, interquartile range and minimum-to-maximum values; ^†^*p*=0.05, *p<0.05, **p<0.01. Tx, treatment

No participant was free of SHEs in the year before transplant (Table 2), and hypoglycaemia severity was significantly reduced in both groups within the first month post transplant and maintained throughout the study. HYPO scores were significantly different between cohorts only at 24 months post transplantation (Fig. 2a).

Following transplantation, 15 participants (65%) receiving tac/MMF and nine participants (100%) receiving bela/siro had measurable (>0.05 nmol/l) fasting C-peptide at 12 months (*p*=0.069, Fisher’s exact test). The numbers dropped to 13 (65%) and eight (89%), respectively, at 24 months (*p*=0.37). Fasting C-peptide was significantly higher in the bela/siro group at 6 months post transplant (Fig. 2b). The highest documented fasting C-peptide was not significantly higher in the bela/siro group (0.43±0.31 vs 0.62±0.3 nmol/l, *p*=0.061) and occurred later (77.4±56.7 vs 214±147 days, *p*=0.0024), although the number of transplants was not significantly different (*p*=0.20). Mean HbA_1c_, elevated at baseline [68.4±16.3 in the tac/MMF group vs 60.8±12.6 mmol/mol in the bela/siro cohort (8.4±1.5% vs 7.7±1.5%), *p*=0.19], fell to 47.8±14.9 vs 54.1±0.7.2 mmol/mol (6.5±1.1% vs 6.8±0.68%, *p*=0.118) and 51.1±13.4 vs 56.8±9.9 mmol/mol (6.8±1.2% vs 7.3±0.92%, *p*=0.237) at 12 and 24 months, respectively (Fig. 2c).

Insulin requirements at baseline were equivalent (0.53±0.24 vs 0.47±0.1 U kg^−1^ day^−1^, *p*=0.29) and decreased dramatically following transplantation. There was significantly lower insulin dosing at 1, 3 and 6 months in the bela/siro group (Fig. 2d), and sustained reductions at 12 and 24 months post transplant in both groups compared with baseline. Six of the 24 individuals (25%) receiving tac/MMF and three (33%) receiving bela/siro were insulin independent (≤5 U/day) within 12 months. Time to insulin independence was not significantly different between groups (127±159 vs 43.5±13.6 days, *p*=0.15), nor was its duration (619±652 vs 453±160 days, *p*=0.79).

### Sensitisation to HLA

Participants in the tac/MMF cohort demonstrated panel reactive antibodies (PRA) ranging from 0% to 30% at the time of first islet transplant, compared with 0% to 60% in the bela/siro group. Sixteen (67%) tac/MMF-treated individuals demonstrated multiple de novo class I and/or class II donor-specific antibodies (DSA), developing at 302±423 days (range 13–1476) post transplantation, and with peak mean fluorescence intensity (MFI) 3989 (range 623–16,956). Four participants (44%) from the bela/siro cohort developed a more limited range of DSA at 299±359 days (range 14–905) at lower titre (*p*=0.091, Mann–Whitney *U* test). Of the bela/siro cohort, three of the four participants had transient expression of DSA and only one had a persisting DSA, whereas in the tac/MMF cohort ten of the 16 participants had persistent DSA. The results are summarised in ESM Table 3.

### Adverse events

There were no deaths within the study time. Three individuals from the tac/MMF group experienced a major adverse cardiovascular event in the peri-transplant period, with two requiring percutaneous coronary intervention.

All participants received post-infusion heparin for 24 h, and no ultrasonographic evidence of complete portal thrombosis was documented. Three individuals had radiological evidence of partial portal vein thrombosis which resolved with formal anticoagulation. Procedure-related complications included four post-operative bleeds, two requiring transfusion (all receiving tac/MMF).

Six individuals (67%) commenced on bela/siro developed mouth ulcers compared with one (out of 22, 5%) in the tac/MMF cohort. Two participants were switched to alternative immunosuppression (bela/MMF) after 12 months due to side effects, and three were changed to bela/tac (two for the third transplant, one for fertility issues). One participant from the bela/siro cohort discontinued belatacept (at day 675) for convenience and was converted to tac/siro. Adverse events are summarised in ESM Table 4.

**Fig. 3:**
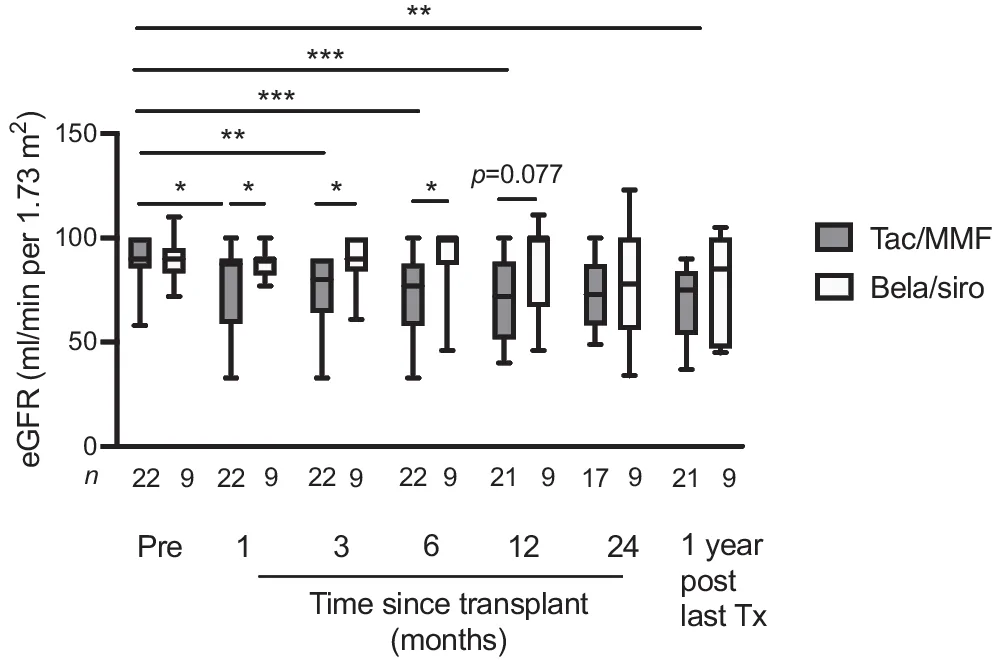
Renal function after islet transplantation. eGFR pre and post islet transplantation (1, 3, 6, 12 and 24 months after the first islet transplant and 12 months after the last transplant) as calculated by the CKD-EPI (Chronic Kidney Disease Epidemiology Collaboration) equation in participants receiving bela/siro or tac/MMF immunosuppression. Longitudinal and between-group comparisons were performed using a mixed-model effect. Results are reported as median, interquartile range and minimum-to-maximum values; *p<0.05, **p<0.01, ***p<0.001. Tx, treatment

Three individuals in the tac/MMF group also experienced an episode of acute kidney injury during the study period. There was also a significant decline in estimated glomerular filtration rate (eGFR) in the tac/MMF group from baseline to all timepoints post transplantation (Fig. 3), with the greatest nadir at 12 months (mean 90.5 ml/min pre-transplant compared with 69.7 ml/min, *p*<0.0001). Twelve participants (50%) in the tac/MMF group had a >20% reduction in eGFR, compared with one participant (10%) from the bela/siro group.

### Immunophenotyping

To assess longitudinal changes in the peripheral immune cell population associated with immunosuppression, we analysed whole blood from cross-sectional, freshly isolated samples. We first ascertained absolute numbers of circulating CD14^+^ monocytes, CD56^+^ NK cells, CD56^+^CD3^+^ NKT cells, CD19^+^ B cells and CD3^+^ T cells (ESM Fig. 1), demonstrating substantial, persistent reductions following induction immunosuppression, but no difference between cohorts with the exception of CD19^+^ B cells.

**Fig. 4:**
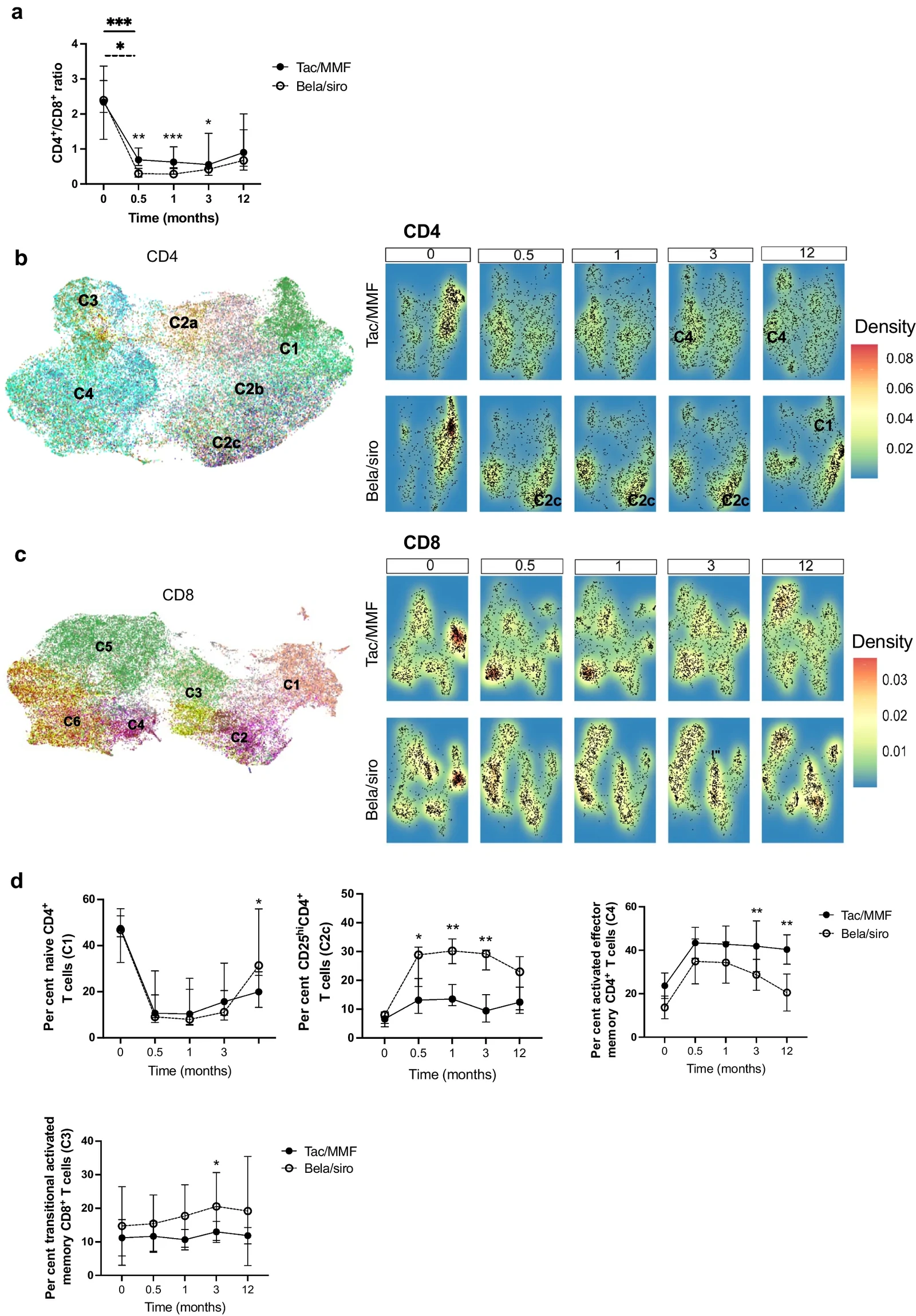
Longitudinal patterns in CD4^+^ and CD8^+^ T cell populations in islet transplant recipients receiving either bela/siro or tac/MMF. (**a**) CD4⁺/CD8⁺ T cell ratio comparison across time in the tac/MMF (*n*=13) and bela/siro (*n*=7) groups. UMAP identification of (**b**) CD4^+^ and (**c**) CD8^+^ T cell clusters based on the expression of CD45, CD45RA, CD3, CD25, CD127, CD62L, CCR7, CD4 and/or CD8 within gated CD4⁺CD3⁺ and CD8⁺CD3⁺ T cells, respectively. UMAPs for CD4⁺ and CD8⁺ T cells were separately generated. CD4⁺ T cell clusters were defined as follows: C1: naive; C2a: early central memory (CM-1, CD62L⁺, CCR7⁻); C2b: central memory (CM1–2, CD62L⁺, CCR7⁺); C2c: CD25^high^ (containing Tregs); C3: transitional activated memory (referred to as activated memory CD62L^+/low^, heterogeneous CD45RA, CD127^−/low^, CCR7^−^); and C4: activated effector memory (EM2, CD25^+^). CD8⁺ T cell clusters were defined as follows: C1: naive; C2: central memory; C3: transitional activated memory; C4: central memory-like/trafficking; C5: terminally differentiated effector memory (TEMRA); and C6: effector memory. Longitudinal patterns of CD4⁺ and CD8⁺ T cells were visualised on UMAPs in the tac/MMF (*n*=13) and bela/siro (*n*=5) groups. Clusters with significant differences between groups are labelled. Longitudinal analyses of UMAP-defined (**d**) CD4⁺ and (**e**) CD8⁺ T cell subsets. Results are reported as median ± interquartile range; *p<0.05, **p<0.01, ***p<0.001

Next, we performed unsupervised high-dimensional analyses of T and B cell panels to create consensus UMAPs across time and treatment groups. The ratio of CD4^+^ to CD8^+^ T cells was inverted following administration of ATG, and significantly lower in the bela/siro group until 12 months (Fig. 4a). UMAP analysis identified four CD4⁺ T cell clusters (comprising six subclusters) (Fig. 4b) and six CD8⁺ T cell clusters (Fig. 4c) based on the expression of CD45, CD45RA, CD3, CD25, CD127, CD62L, CCR7, CD4 and/or CD8 within gated CD4⁺CD3⁺ and CD8⁺CD3⁺ T cells, respectively. Longitudinal analyses of UMAP-defined T cell subsets identified three CD4⁺ T cell clusters and one CD8⁺ T cell cluster with significant differences between treatment groups. The proportion of naive CD4⁺ T cells (C1) among total CD4⁺ T cells was significantly higher at 12 months in the bela/siro group compared with tac/MMF. CD25^high^CD4⁺ T cell (Treg-like; C2c) proportions were significantly higher at the 0.5, 1 and 3 month timepoints in the bela/siro group, while activated effector memory CD4⁺ T cells (C4) were significantly higher at 3 and 12 months in the tac/MMF group (Fig. 4d). The proportion of transitional activated memory CD8^+^ T cells (C3) was significantly higher at 3 months post transplant in the bela/siro group (Fig. 4e).

**Fig. 5:**
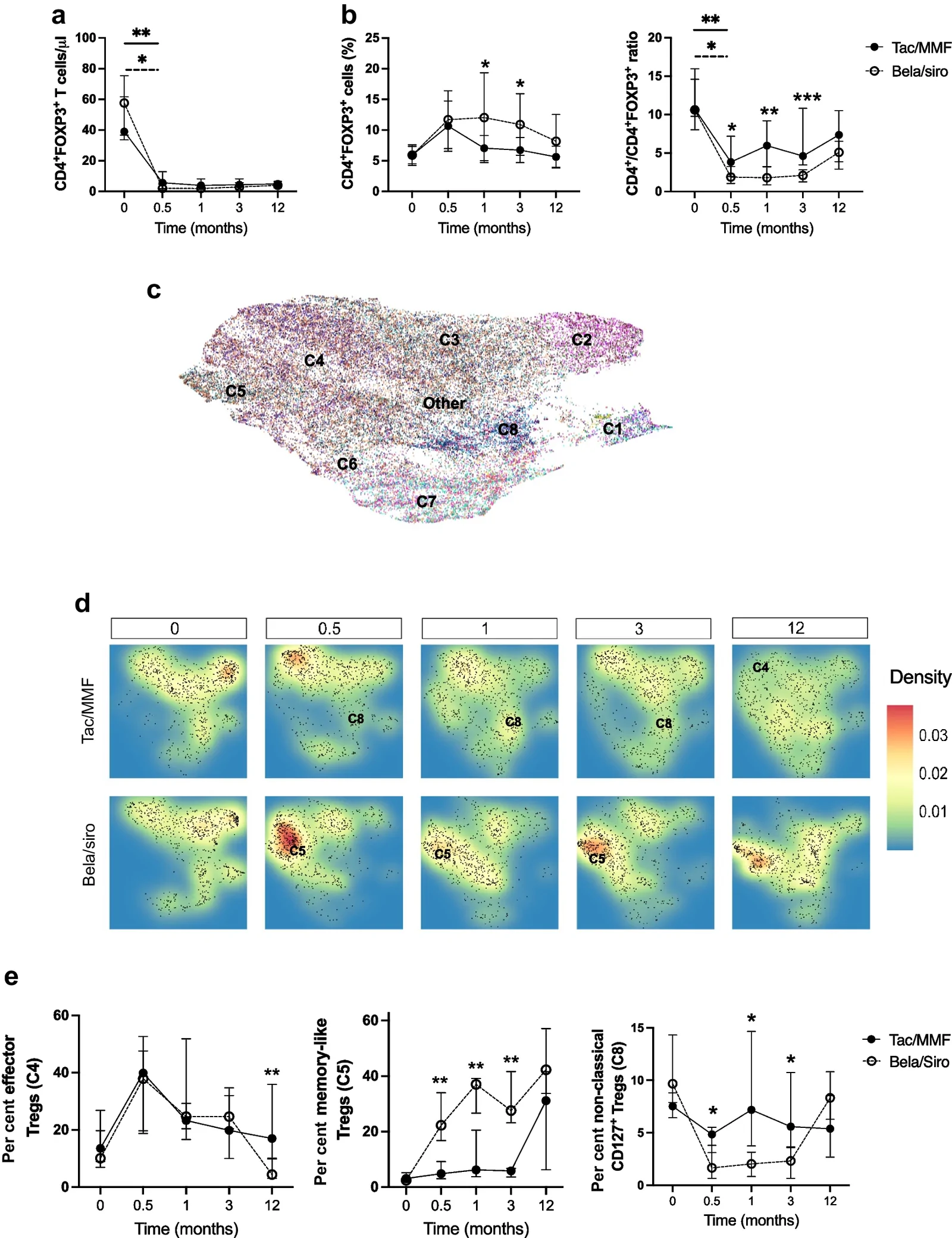
Longitudinal patterns in Treg populations in islet transplant recipients receiving either bela/siro or tac/MMF. (**a**) Total CD4⁺FOXP3⁺ Treg count. (**b**) Proportion of CD4⁺FOXP3⁺ cells among CD4⁺ T cells and CD4⁺/CD4⁺FOXP3⁺ T cell ratio over time. (**c**) UMAP identification of Treg clusters based on the expression of CD45RO, CD127, CD25, CD39, CD137, CD154 and FOXP3 within gated CD45⁺CD3⁺CD4⁺FOXP3⁺ Tregs. The identified subclusters were: C1: naive-like (CD25^dim^ Tregs); C2: naive (CD25^bright^ Tregs); C3: naive-like transitional Tregs (CD45RO^dim^CD39^−^); C4: effector Tregs (CD39⁺CD45RO⁺); C5: memory-like Tregs (CD39⁻CD45RO⁺); C6: activated CD39⁺CD25^dim^ Tregs (CD45RO heterogeneous); C7: CD25⁻ memory-like Tregs; C8: non-classical memory Tregs (CD127⁺CD45RO⁺); and other: un-classified cells. (**d**) Longitudinal patterns of Treg subsets visualised on UMAP. (**e**) Longitudinal analyses of UMAP-defined effector Tregs (C4), memory-like Tregs (C5) and non-classical CD127^+^ Tregs (C8). Results are reported as median ± interquartile range; *p<0.05, **p<0.01, ***p<0.001 as shown (dashed brackets Bela/siro, solid brackets Tac/MMF)

A similar analysis was performed for CD4^+^FOXP3^+^ Tregs. Although there was no difference in absolute cell numbers (Fig. 5a), the proportion of CD4⁺FOXP3^+^ Tregs was higher in the bela/siro group at 1 and 3 months, and the ratio of CD4⁺/CD4⁺FOXP3⁺ cells was lower (Fig. 5b). Nine Treg subclusters (Fig. 5c) were identified by UMAP based on expression of CD45RO, CD127, CD25, CD39, CD137, CD154 and FOXP3 within gated CD45⁺CD3⁺CD4⁺FOXP3⁺ Tregs. When followed longitudinally (Fig. 5d), we identified significantly elevated proportions of effector Tregs (C4) at the 12 month timepoint in the tac/MMF group (Fig. 5e). The proportion of memory-like Tregs (C5) was significantly higher in the bela/siro group at 0.5, 1 and 3 months post transplant. In contrast, the proportion of non-classical CD127^+^ Tregs (C8) was significantly higher in the tac/MMF group at the same timepoints.

Eight B cell clusters were identified by UMAP (ESM Fig. 2a) based on differential expression of IgM, IgG, CD27, CD38, CD24 and CD21 within gated CD45⁺CD19⁺ B cells. Longitudinal analyses (ESM Fig. 2b) demonstrated significantly higher proportions of transitional B cells including Breg-like cells (C3) in bela/siro participants at 3 months (ESM Fig. 2c). The proportion of atypical class-switched memory B cells (C6) was also significantly higher at 0.5 and 3 months in the bela/siro group.

## Discussion

This prospective study explored the efficacy of bela/siro immunosuppression compared with a CNI-based standard of care. Superior glucose levels were achieved with bela/siro compared with the CNI protocol, particularly at early timepoints post transplantation (1–3 months). There was striking improvement in beta cell function early after transplantation in the bela/siro group, contributed to by the deleterious impact of CNIs on islet function. Fewer transplants were required to reach insulin independence in the bela/siro cohort, although the effect was not necessarily durable. Importantly, renal function was not compromised by CNI-free immunosuppression, with stable eGFR to 24 months after the first islet transplant. The study presented here again confirms the utility of clinical islet transplantation in reducing hypoglycaemic events in individuals with type 1 diabetes with severe hypoglycaemia and metabolic instability, even with modest C-peptide levels. For the first time we also comprehensively report longitudinal changes in immune cell populations in peripheral blood, demonstrating variations in regulatory T and B cell populations following treatment with bela/siro, which may account for the improved metabolic response to islet transplantation observed, as well as lower frequency and titre of DSAs (a secondary outcome of the study).

Belatacept is a selective T cell co-stimulation blocker, consisting of the extracellular domain of cytotoxic T-lymphocyte antigen 4 (CTLA4) coupled to the Fc-tail of IgG. Co-stimulatory blockade is an effective immunomodulatory approach that limits the CD80/86 signalling required for full activation of alloreactive T cells. It can dramatically prolong vascularised allograft survival [20], with superior long-term function compared with treatment with CNIs in renal allografts [6]. Preclinical data suggest that mTOR inhibitors might provide synergistic immunosuppression when coupled with co-stimulation blockade. The use of bela/siro in NHP allo-islet transplantation [21] resulted in immediate graft function without alloantibody production, suggesting a role in maintenance immunosuppression that avoids the diabetogenic effect of CNIs. In addition, belatacept has not been shown to affect islet viability or insulin secretion [22], and conversion to co-stimulatory blockade in diabetic kidney allograft recipients substantially improves glycaemic parameters [23].

Few studies have robustly characterised leukocyte subsets associated with belatacept treatment. Previous single-centre, small cohort studies in kidney transplant recipients have demonstrated lower numbers of B and T lymphocytes as well as CD14-negative monocytes in individuals treated with belatacept compared with CNIs [24]. Belatacept did not impair the recovery of B, T and NK cell populations after initial depletion following thymoglobulin induction, although observations were made on only a subset of individuals, and there was no long-term effect on circulating Tregs. Moreover, belatacept-treated individuals have been shown to have significantly greater numbers of FOXP3^+^ Tregs in graft biopsies during acute rejection compared with CNI-treated individuals [25]. In this study we clearly demonstrate higher proportions of native CD4^+^ T cells, CD25^+^ Tregs and memory-like Tregs in the bela/siro cohort, suggesting early, superior immunoregulation. These data are in keeping with known preservation of IL-2 signalling by belatacept and calcineurin-medication Treg inhibition. Belatacept also blocks CD28-mediated activation of T follicular helper cells, reducing B cell-driven activation and antibody responses [26], and our data support modulation of humoral immunity and protection against DSA formation. This was complemented by increased activated effector memory CD4^+^ T cells in the tac/MMF group, and expansion of transitional B cell subsets, including Breg-like cells, in the bela/siro cohort.

Several studies have reported that sirolimus, but not CNIs, contributes to the generation of high numbers of CD4^+^FOXP3^+^ Tregs in vitro and that these Tregs can be expanded without losing their suppressive activity and Foxp3 expression [12]. Selective expansion of functional CD4^+^FOXP3^+^ Tregs, crucial for the development and maintenance of tolerance, and increased CD4^+^CD45RA^−^CCR7^−^ effector memory T cells have also been described [27]. Due to its negligible nephrotoxicity and capacity to harness tolerogenic pathways, sirolimus remains an attractive alternative to CNIs in non-renal transplantation where recipients demonstrate a residual risk of chronic kidney disease [5]. However, sirolimus also disrupts glucose homeostasis by promoting insulin resistance [28] and beta cell dysfunction [29]. Until a less restricted source of donor islets is readily available, through allogeneic [30] or autologous chemically induced pluripotent [31] stem-cell-derived beta cells, the focus will remain on optimising immunosuppression to improve islet function.

This open-label study has several limitations, including the small sample size (which increases the potential for type II error and limited the ability to assess potential sex- or gender-related differences), relatively slow recruitment and lengthy range of dates across which participants received islet transplants. The COVID-19 pandemic affected recruitment, treatment and accessibility of this multi-centre trial, with the first islet transplant for the bela/siro arm performed in June 2019. The Australian Islet Transplant Consortium provides a nationwide islet transplant programme. The implementation of differing, and frequently changing, state-based public health directives, particularly travel restrictions, lockdowns and border closures, had a marked impact on study operations in the islet isolation and transplant centres, as many of the participants were required to travel interstate for treatment. These restrictions led to interruptions in participant recruitment and screening, lower-than-anticipated enrolment and delays in treatment. Enrolled participants experienced significant barriers in attending study visits, with mandatory quarantines and abrupt lockdowns leading to missed appointments. Interstate pandemic-related socioeconomic pressures further reduced participant retention and adherence. Given that the long-term success of islet transplantation depends heavily on supplementing beta cell mass, the lower islet cell mass and longer timeframes between successive transplants in the bela/siro cohort may well have affected rates of insulin independence and graft longevity, despite the early benefits demonstrated.

In summary, this pivotal phase 2 trial in individuals with type 1 diabetes and IAH shows that T cell depletion with ATG in combination with bela/siro provided good anti-rejection cover in islet transplantation, was well tolerated by participants and supported better beta cell function compared with the CNI-based regimen. Rates of insulin independence were comparable to standard of care (using tacrolimus and MMF). Our data also demonstrated attenuation of immune effector cell responses, supporting the biological rationale for the use of bela/siro in islet transplantation, although additional studies are needed to extend the mechanistic understanding in relation to clinical outcomes and evaluation of different immunosuppressive regimens for this cellular therapy.

## Electronic supplementary material

Below is the link to the electronic supplementary material.

Supplementary file1 (PDF 908 KB)

## Data Availability

Individual participant data will not be made available. The study protocol, statistical analysis plan and analytical code will be available upon publication in response to any reasonable request to the corresponding authors.

## Appendix

Australian Islet Transplant Consortium

Westmead Hospital, Western Sydney Local Health District: P. Anderson, M. Azar, H. Burns, Y. Chew, K. Ghimire, W. J. Hawthorne, J. Joo, N. Jozan, L. Williams

St Vincent’s Hospital: M. Damasiewicz, K. Howe, F. Ierino, G. Parsons

Royal Adelaide Hospital: T. Radford, A. Rickard

## Abbreviations

ATG: Antithymocyte globulin
Bela/siro: Belatacept/sirolimus; belatacept in combination with sirolimus immunosuppression
CNI: Calcineurin inhibitor
DSA: Donor-specific antibodies
eGFR: Estimated glomerular filtration rate
GAD65: Glutamic acid decarboxylase-65
IA-2: Islet antigen-2
IAH: Impaired awareness of hypoglycaemia
IEQ: Islet equivalents
mTOR: Mammalian target of rapamycin
SHE: Severe hypoglycaemic events
Tac/MMF: Tacrolimus/mycophenolate mofetil
Treg: Regulatory T cell
UMAP: Uniform Manifold Approximation and Projection
