# Supplementary material for: A multi-centre study of belatacept and sirolimus for islet transplantation
Source: Diabetologia. 2026 Aug 17;69(10):2847–61. doi: 10.1007/s00125-026-06813-3 (PMC13541912; doi:10.1007/s00125-026-06813-3)
Supplement: Supplementary file 1 [file 125_2026_6813_MOESM1_ESM.pdf]

ESM Table 1 Immunosuppression

**Induction immunosuppression**	
Anti-thymocyte globulin<br>1st transplant<br>2nd transplant	 3.0mg/kg on Day 1, 1.5mg/kg on Day 5<br>1.5mg/kg on Day 1, Day 5
Basiliximab	20mg IV on Day 0 (3rd transplant only)
Etanercept (Tac/MMF recipients only)	50mg IV on Day 0<br>25mg IV on Day 3, 7, 10
**Maintenance immunosuppression**	
Tacrolimus	0.1 mg/kg/day in two divided doses<br>Trough level 10-12 ng/ml with MMF<br>Trough level 5-6 ng/ml with sirolimus
Mycophenolate mofetil (MMF)	1g PO bd
Sirolimus	5mg daily<br>Trough level 5-8 ng/ml
Belatacept<br>1st transplant<br><br><br>2nd/3rd transplant	 10mg/kg Day 1 and 5<br>10mg/kg Weeks 2, 4, 8 and 12<br>5mg/kg monthly thereafter<br>5mg/kg Day 1 and 5<br>5mg/kg Weeks 2, 4, 6 and 8<br>5mg/kg monthly from week 12
Metformin (all islet transplant recipients)	1g PO daily
Sitagliptin (all islet transplant recipients)	100mg daily PO daily

Abbreviations: PO – per oral

ESM Table 2 Missing Variables

Clinical Parameter	Tac/MMF	Bela/siro
c-peptide		
pre-transplant	0	0
post 1st islet transplant		
1 mo	0	0
3 mo	0	0
6 mo	0	0
12 mo	0	0
24 mo	0	1
12 mo post last transplant	0	0
HYPO		
pre-transplant	1	1
post 1st islet transplant		
1 mo	6	4
3 mo	8	3
6 mo	3	2
12 mo	2	0
24 mo	1	2
12 mo post last transplant	3	3
Insulin dose U/kg		
pre-transplant	0	0
post 1st islet transplant		
1 mo	0	0
3 mo	0	0
6 mo	0	0
12 mo	0	0
24 mo	1	0
12 mo post last transplant	2	0
HbA1c		
pre-transplant	0	0
post 1st islet transplant		
1 mo	2	0
3 mo	1	0
6 mo	0	0
12 mo	0	0
24 mo	0	0
12 mo post last transplant	2	0
eGFR (ml/min/1.73m^2)		
pre-transplant	0	0
post 1st islet transplant		
1 mo	0	0
3 mo	0	0
6 mo	0	0
12 mo	0	0
24 mo	2	0
12 mo post last transplant	0	0

ESM Table 3 de novo DSA following islet transplantation.

	De novo DSA	MFI – highest titre	Time to DSA (days)	Persistent
Tac/MMF				
Class I				
M17	Y (multiple)	1265	318	Y
M18	Y (multiple)	2866	26	N
M19	Y (multiple)	979	209	N
M21	Y (multiple)	7252	209	N
M22	Y	3666	13	N
WM16	Y	1133	17	Y
WM17	Y	1665	1476	N
WM21	Y	854	32	N
SANTIP06	Y	623	30	?
SANTIP07	Y (multiple)	1316	250	N
SANTIP08	Y	16956	61	Y
SANTIP09	Y (multiple)	8511	31	Y
Class II				
M14	Y	1497	158	Y
M20	Y	7844	1168	Y
M21	Y (multiple)	1172	988	N
WM16	Y	1609	185	Y
WM21	Y (multiple)	6775	32	Y
SANTIP07	Y (multiple)	10369	250	Y
SANTIP08	Y	2229	61	Y
SANTIP10	Y	4499	764	N
SANTIP16	Y	698	60	N
Bela/siro				
Class I				
WM23	Y	537	43	N
RAH01	Y	1311	266	N
RAH03	Y	1062	14	N
Class II				
WM26	Y	823	905	Y
RAH03	Y	2888	266	N

Abbreviations: Bela/siro – belatacept/sirolimus, cPRA – current panel reactive antibody, DSA – donor-specific antibody, de novo - new onset, HLA – human leukocyte antigen, MFI – mean fluorescence index, tac/MMF – tacrolimus/mycophenolate mofetil.

ESM Table 4 Adverse events

	**Tac/MMF**<br>**N=24**	**Bela/siro**<br>**N=9**
Medication related	Mouth ulcers n=1	Mouth ulcers n=6<br>peripheral oedema n=1 (presumed sirolimus-related)
Bleeding	n=4<br>CVC insertion n=1<br>hepatic n=3	-
Portal vein thrombosis (partial)	n=1	n=2
Major adverse cardiovascular events	n=3<br>2x PCI, 1x CABG	-

Abbreviations: Bela/siro – belatacept/sirolimus, CABG – coronary artery bypass grafting, CVC – central venous catheter, PCI – percutaneous coronary intervention, tac/MMF – tacrolimus/mycophenolate mofetil

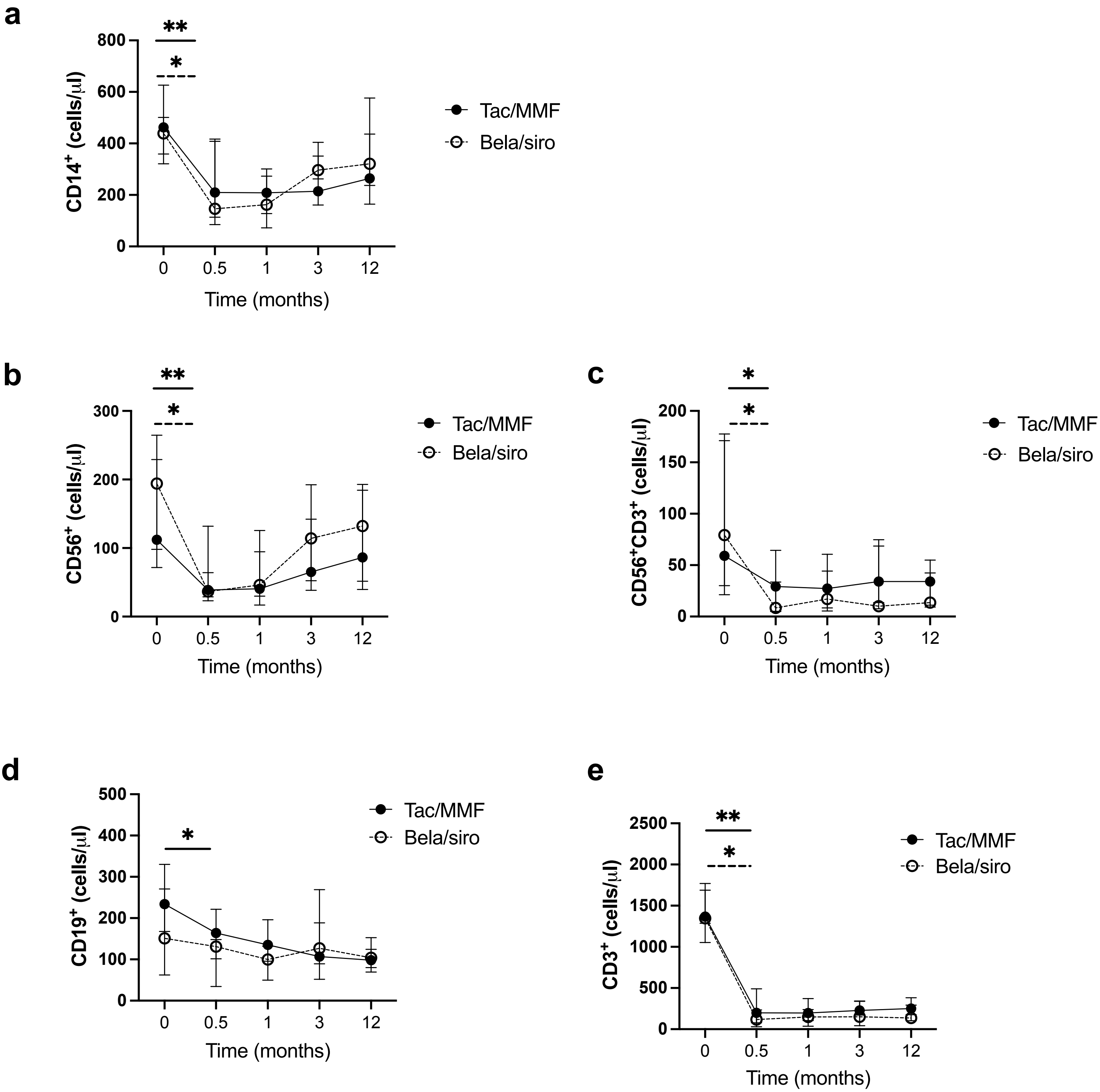

ESM Figure 1

Changes in immune cell numbers in peripheral blood following Bela/siro or Tac/MMF immunosuppression for islet transplantation.

Blood samples were taken prior to transplantation (pre), and at 0·5, 1, 3, and 12 months post-islet transplantation. (a) $CD14^+$ monocytes, (b) $CD56^+$ NK cells, (c) $CD56^+CD3^+$ NKT cells, (d) $CD19^+$ B cells, and (e) $CD3^+$ T cells in the tac/MMF (n = 13) and Bela/siro (n = 7) groups. Results are reported as median interquartile range, *p<0.05, **p<0.01.

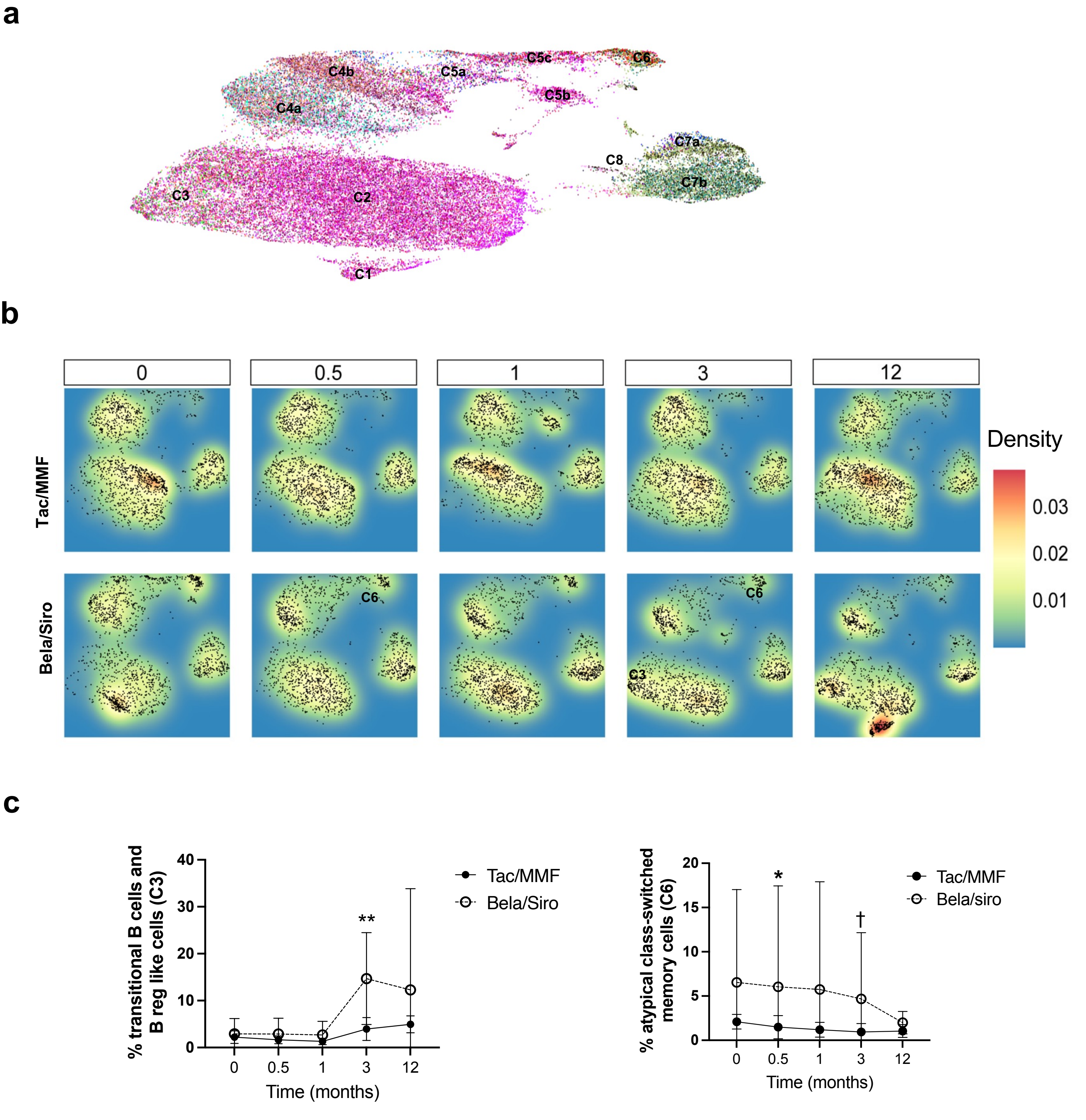

ESM Figure 2

Longitudinal patterns of B cell populations in islet transplant recipients receiving either Bela/siro or tac/MMF.

(a) UMAP identification of B cell clusters based on differential expression of IgM, IgG, CD27, CD38, CD24, and CD21 within gated $CD45^{+}CD19^{+}$ B cells. The identified clusters are as follows: C1: Naïve B cells (resting), C2: Naïve B cells, C3: Transitional B cells containing Breg-like (regulatory B cell-like). C4a: non-switched memory (NSM, activated), C4b: NSM (resting), C5a: mature naïve B cells, C5b: early activated naïve B cells, C5c: activated/atypical B cells, C6: atypical class-switched memory (aCSM), C7a: classical switched memory (CSM, resting), C7b: CSM (activated), C8: plasmablasts.

(b) Longitudinal patterns of B cell subsets visualized on UMAP.

(c) Longitudinal analyses of B cell subsets with statistical comparisons corresponding to UMAP-defined clusters. The proportion of transitional B-cells including B-reg like cells (C3) and atypical class-switched memory B-cells (C6) among total B cells.

Results are reported as median interquartile range, † $p=0.05$, * $p<0.05$, ** $p<0.01$.
